# A three-dimensional approach to visualize pairwise morphological variation and its application to fragmentary palaeontological specimens

**DOI:** 10.7717/peerj.10545

**Published:** 2021-01-19

**Authors:** Matt A. White, Nicolás E. Campione

**Affiliations:** 1 Palaeoscience Research Centre, School of Environment and Rural Science, University of New England, Armidale, NSW, Australia; 2 Palaeontology, Australian Age of Dinosaurs Museum of Natural History, Winton, QLD, Australia

**Keywords:** Australovenator, NMV P186153, Megaraptorid, Pairwise

## Abstract

Classifying isolated vertebrate bones to a high level of taxonomic precision can be difficult. Many of Australia’s Cretaceous terrestrial vertebrate fossil-bearing deposits, for example, produce large numbers of isolated bones and very few associated or articulated skeletons. Identifying these often fragmentary remains beyond high-level taxonomic ranks, such as Ornithopoda or Theropoda, is difficult and those classified to lower taxonomic levels are often debated. The ever-increasing accessibility to 3D-based comparative techniques has allowed palaeontologists to undertake a variety of shape analyses, such as geometric morphometrics, that although powerful and often ideal, require the recognition of diagnostic landmarks and the generation of sufficiently large data sets to detect clusters and accurately describe major components of morphological variation. As a result, such approaches are often outside the scope of basic palaeontological research that aims to simply identify fragmentary specimens. Herein we present a workflow in which pairwise comparisons between fragmentary fossils and better known exemplars are digitally achieved through three-dimensional mapping of their surface profiles and the iterative closest point (ICP) algorithm. To showcase this methodology, we compared a fragmentary theropod ungual (NMV P186153) from Victoria, Australia, identified as a neovenatorid, with the manual unguals of the megaraptoran *Australovenator wintonensis* (AODF604). We discovered that NMV P186153 was a near identical match to AODF604 manual ungual II-3, differing only in size, which, given their 10–15Ma age difference, suggests stasis in megaraptoran ungual morphology throughout this interval. Although useful, our approach is not free of subjectivity; care must be taken to eliminate the effects of broken and incomplete surfaces and identify the human errors incurred during scaling, such as through replication. Nevertheless, this approach will help to evaluate and identify fragmentary remains, adding a quantitative perspective to an otherwise qualitative endeavour.

## Introduction

Vertebrate fossil collections are fraught with incomplete specimens that are difficult to compare morphologically, thereby making their taxonomic and anatomical identities difficult, if not impossible to assess. Ideally, morphometric techniques such as geometric morphometrics should be used to estimate missing data and place incomplete specimens within the context of a morphospace (Claude, 2008; O’Higgins et al., 2011; Zelditch, Swiderski & Sheets, 2012; Arbour & Brown, 2014; Rohlf & Slice, 1990; Adams, Rohlf & Slice, 2013; Piras et al., 2014; Pieterse, Benítez & Addison, 2017; Polly & Head, 2004; Cope et al., 2012; Demayo, Harun & Torres, 2011). However, morphometrics require: (1) the recognition of diagnostic landmarks, (2) the generation of large data sets to detect clusters and accurately describe major components of morphological variation, and, (3) in the case of missing data estimation, that the available landmarks are sufficiently and adequately sampled. As a result, such approaches are often outside the scope of fundamental palaeontological research that aims to identify and interpret fragmentary specimens from geographic regions and temporal time-intervals known to produce important but fragmentary specimens, such as that of the Australian Mesozoic terrestrial fossil record (Von Huene, 1932; Agnolin et al., 2010; White et al., 2013, 2020; Molnar & Pledge, 1980; Molnar, Flannery & Rich, 1981; Barrett, Kear & Benson, 2010; Rich & Vickers-Rich, 1994, 2003; Benson et al., 2010a, 2010b, 2012; Herne, Nair & Salisbury, 2010; Rich et al., 2014; Poropat et al., 2018, 2019; Long & Molnar, 1998; Fitzgerald et al., 2012; Brougham, Smith & Bell, 2019; Bell et al., 2016).

The accessibility of 3D visualisation techniques, such as computed tomography, 3D surface scanning and mesh manipulation software (e.g. Zbrush—Pixologic Inc., Los Angeles, CA, USA), along with the development of a suite of open-source software (e.g. MeshLab, CloudCompare, 3D Slicer) has enabled users to feasibly generate, process, manipulate, and virtually restore fossils for downstream analyses. These advances have led to an ever-increasing sample of open-source 3D reconstructions (e.g. digimoprh.org, phenome10k.org, and others). In palaeontology, 3D modelling is now readily used to conduct biomechanical analyses, visualise internal spaces/structures, and explore morphospace dynamics (Rayfield, 2005; Evans, Ridgely & Witmer, 2009; Hedrick & Dodson, 2013). However, its accessibility suggests that it could be extended to assist with more fundamental questions, such as specimen identification and pairwise comparisons that, in the absence of large data sets, can be used to quantitatively and visually interpret regions of variation. Subsequently, we describe a workflow in which three-dimensional surface profiles of fragmentary fossils (target specimens) can be quantitatively compared to better-known exemplars (reference specimens) via the iterative closest point (ICP) algorithm (Besl & McKay, 1992; Chetverikov et al., 2002). Our aim is to present the potential utility of such an approach to assist with basic palaeontological research and curatorial identification, but stress that its utility is still dependent on the nature and size of the comparative sample.

## Methods and Workflows

### Experimental basis

**Figure 1:**
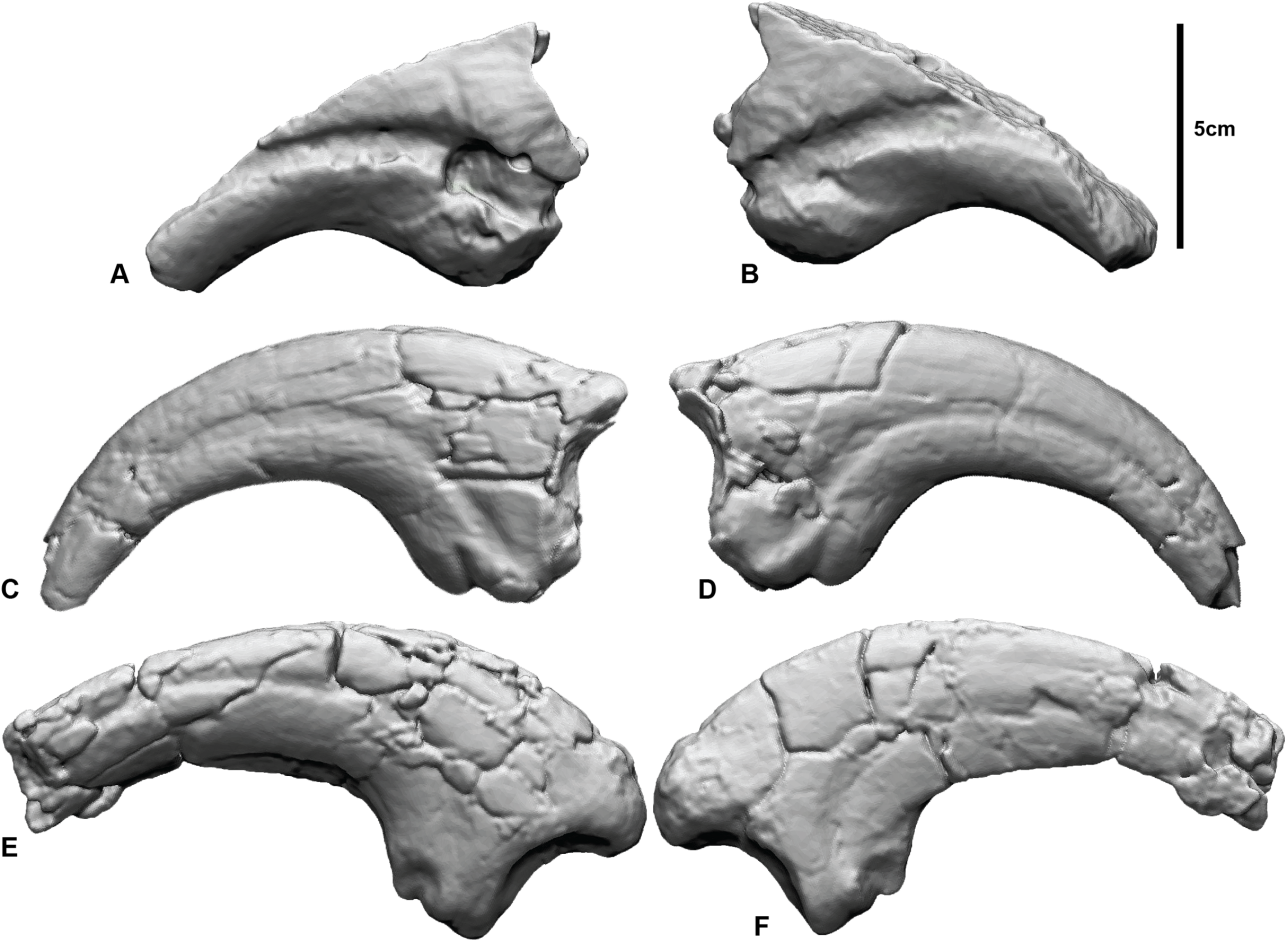
Megaraptorid manual unguals from the Cretaceous of Australia. (A and B) Megaraptorid right manual ungual (NMV P186153) in: (A) lateral, (B) medial. *Australovenator wintonensis* manual ungual II-3 (AODF 604) (C) lateral, (D) medial. *Australovenator wintonensis* manual ungual I-2 (AODF 604) (E) lateral, (F) medial.

To showcase our approach, we revisit a fragment of a large manual ungual, NMV P186153, discovered near Kilcunda, Victoria, Australia, considered to be Australia’s largest theropod (Poropat et al., 2019) (Fig. 1). NMV P186153 was originally assigned to the theropod clade Neovenatoridae (Benson et al., 2012) but was more recently referred to Megaraptoridae (Poropat et al., 2019). Based on an abundance of shed teeth, megaraptorids are considered to be the dominant Australian Cretaceous theropod, despite being known only from three partial skeletons (Hocknull et al., 2009; White et al., 2012, 2013, 2015, 2016, 2020; Bell et al., 2016; Poropat et al., 2019). To date, other Australian Cretaceous theropods do not preserve their manual unguals (Benson et al., 2012; Brougham, Smith & Bell, 2019, 2020) and, since NMV P186153 was recently assigned to Megaraptoridae based on comprehensive comparisons with the megaraptorid *Australovenator* (Poropat et al., 2019), we adopt our new workflow to explore the morphology of NMV P186153 within the context of the manual phalanges I-2 and II-3 in *Australovenator wintonensis* (Fig. 1). A larger dataset was not sought on this occasion, as the main aim of this manuscript was to demonstrate the methodological implementation. However, the approach is certainly amenable to larger samples, which would evidently lead to greater interpretive power. We chose the aforementioned reference specimens due to the evident similarities between NMV P186153 and II-3 and evident dissimilarities with I-2.

### Specimens

All three specimens were previously described (White et al., 2012, 2015; Benson et al., 2012; Poropat et al., 2019) and we only provide a basic anatomical review here. NMV P186153 is a partial manual ungual that preserves the flexor tubercle region, portions of the medial and lateral blood grooves, and roughly a quarter of the original ventral surface. The proximal articular facet and roughly half of the dorsal height of the specimen is missing. There is also a circular excavation artefact at the proximal extremity of the medial blood groove. AODF604 MCII-3 is a near-complete right manual phalanx with very little surface deformation. Both medial and lateral blood grooves are symmetrical about the sagittal plane of the ungual. The flexor tubercle is rounded and bulbous. AODF604 MC1-2 is near complete. The medial surface is flat, compared to the convex lateral surface. The medial groove runs parallel to the ventral claw curvature and terminates ventral of the distal tip. The lateral groove runs parallel to the dorsal surface and terminates dorsally of the distal tip. The flexor tubercle is narrow, with medial and lateral flexor facets accentuating it from the ventral surface.

### Scan processing

For the pairwise analysis to work, at least two 3D surface meshes of the anatomical models are required: the target model in question, in our case NMV P186153, and the reference model(s) (i.e. *A. wintonensis*; AODF 604). Surface 3D meshes can be generated via three principal methods (Cunningham et al., 2014; Davies et al., 2017), these include: CT scans, processed through specialised segmentation software packages (e.g. 3D Slicer (Fedorov et al., 2012), Amira (Konrad-Zuse-Zentrum für Informationstechnik, Berlin, Germany; Thermo Fisher Scientific, Waltham, MA, USA), AVIZO (Visualization and Data Analysis Group at Zuse Institute, Berlin, Germany), DRAGONFLY (Object Research Systems, Montreal, QC, Canada), MIMICS (Materialise NV, Leuven, Belgium), SPIERS (Palaeoware Design)), 3D surface scans (e.g. Artec (Artec Group, Luxembourg, Luxembourg), Breuckmann (Hexagon Manufacturing Intelligence, Grugliasco, Italia), among numerous other), and photogrammetry (e.g. Agisoft–Agisoft LLC, St. Petersburg, Russia).

Our target and reference specimens (together referred to as showcase specimens) were scanned via standard medical computer tomography (CT) obtained from Queensland X-ray, Mackay Mater Hospital in east-central Queensland using a Philips Brilliance CT 64-slice machine producing 0.9 mm slices. Mimics v10.01 (Materialise HQ, Leuven, Belgium) was used to create three-dimensional meshes of specimens from the CT scans.

The meshes were exported as *stl files and were transformed to *obj format in Rhinoceros v5.0 (Robert McNeel and Associates, Seattle, WA, USA). Specific procedural algorithms vary between software packages, recently reviewed by Lautenschlager (2016), and do not require further explanation here. Importantly, the process of generating the 3D mesh should allow for final exportation to an Object file (*.obj) for subsequent importation into a mesh manipulating software. We used Zbrush (Pixologic Inc, Los Angeles, CA, USA) as our key mesh manipulation software package, however, mesh manipulation can be achieved through a number of other packages, including: BLENDER (Blender Foundation-GNU General Public License), Geomagic Studio (3D Systems, Geomagic Inc, Morrisville, NC, USA), MAYA/MeshMixer (Autodesk Inc., San Rafael, CA, USA; Alias Systems Corporation, Toronto, ON, Canada), MeshLab (ISTI-CNR research centre, GNU General Public License GPL), VG Studio (Volume Graphics, Heidelberg, Germany), as well as some of the aforementioned segmentation software.

### Mesh editing workflow

The following work flow describes the various procedures (some optional) to compare specimens in 3D.

#### Smoothing (Optional)

**Figure 2:**
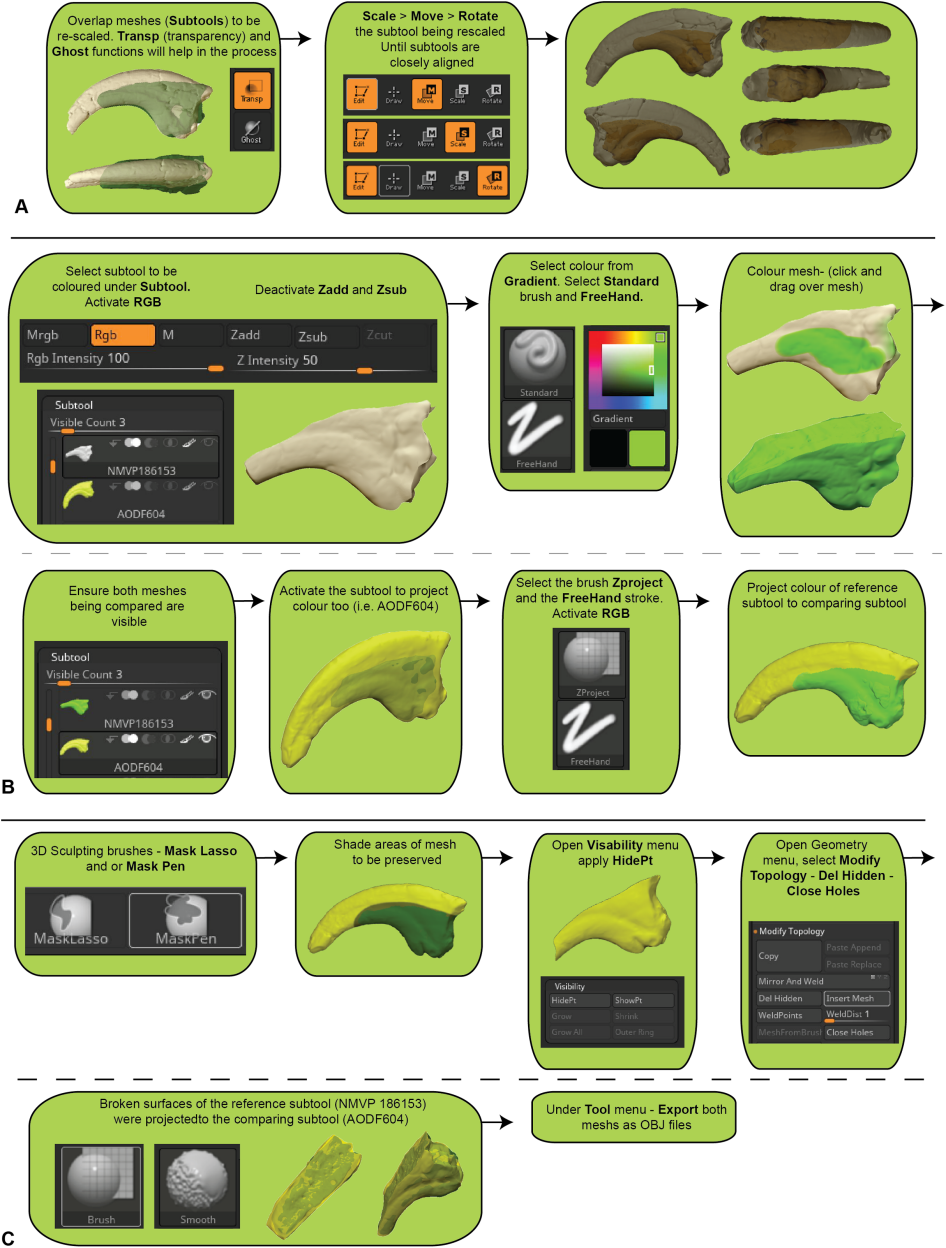
Manual scaling and alignment to prepare digital specimens for pairwise analysis. (A) Workflow of scaling specimens to the same size for pairwise comparisons. (B) Workflow to create a mesh template by projecting the colour from one subtool to another. (C) Workflow for mesh replication using projection tools to replicate fractured edges for pairwise comparisons.

Cracks and voids are a common feature amongst fossils and, whether they are the result of biostratinomic or diagenetic processes, they do not reflect true anatomy. Their removal, through smoothing, may be desirable to limit their effect on final comparisons. In Zbrush (Fig. 2), smoothing is achieved by creating a new base mesh (process detailed in Fig. 2B) and then projecting it onto the original mesh (Fig. 2C). It should be noted that this process will likely lead to a ‘rounding’ of the original scan data, with some loss of resolution. Careful consideration should, therefore, be given to determine whether cracks and minor preservational artefacts could be restored to approximate the original morphology or whether it is better to exclude them (see “Trimming and Replication” section), in which case smoothing is not necessary.

#### First registration

Variation between two fossil specimens, whether biological or taphonomic, is virtually guaranteed, even if they are seemingly identical. For instance, in our showcase specimens, there is considerable size variation between NMV P186153 and reference specimens (AODF604 MCI-2, MCII-3) and there are notably missing regions of the target specimen, such as the entire distal end of the claw. Subsequently, prior to the pairwise comparison, there is need to scale and align (i.e. register) the reference specimens to the target specimen so as to maximise the biological shape variation and minimise ‘noise’ introduced by positional and artefactual variation.

**Figure 3:**
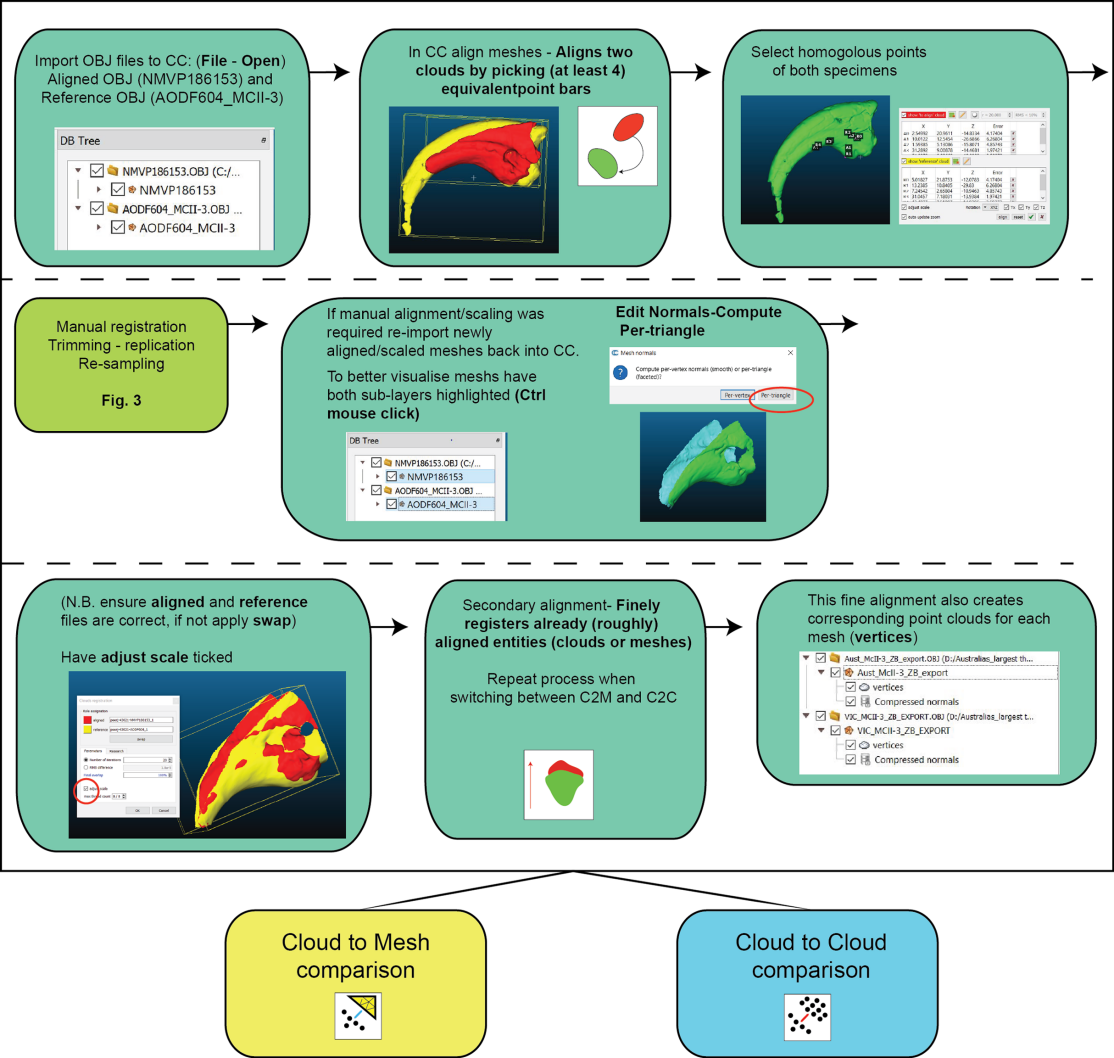
Work flow of importing OBJ files into Cloud Compare and completing first registration alignment and second registration alignment.

Much like geometric morphometrics (as reviewed by Palci & Lee (2018)), first registration requires an initial establishment of homology to justify the comparison. Ideally, homology is based on biological similarity of form and function (i.e. the similarity criterion for primary homology, as defined by De Pinna (1991)). Registration should therefore seek to find structurally and/or topologically similar regions on which to ground scaling and alignment. In our ungual example, the flexor tubercle and both medial and lateral blood grooves were targeted as regions of primary homology and identified via a series of points. The points are then used as the starting point for the ICP algorithm to automatically scale and align the meshes (Fig. 3). Inspection of the scans should follow the process to determine whether the automated process resulted in appropriate scaling and alignment.

#### Manual registration (Optional)

Further manual scaling and aligning may be necessary if the *First Registration* fails to scale and align the specimens adequately. We recommend attempting the *First Registration* alignment numerous times to achieve the best possible alignment with the aim of avoiding a manual registration. If manual registration is required, it can be achieved in Zbrush (Fig. 2A). The ICP algorithm can be later employed following manual alignment to produce more optimally aligned data for pairwise comparisons.

#### Trimming or replication (Optional)

Major areas of variation in the compared specimen interpreted or known to be the result of taphonomy or preparation need to be either removed or duplicated. Our target specimen, for example, is missing its entire distal end and there is an excavation artefact present at the proximal extremity of the medial blood groove. If ignored, these regions would present as major areas of uninformative variation (Fig. 2).

**Figure 4:**
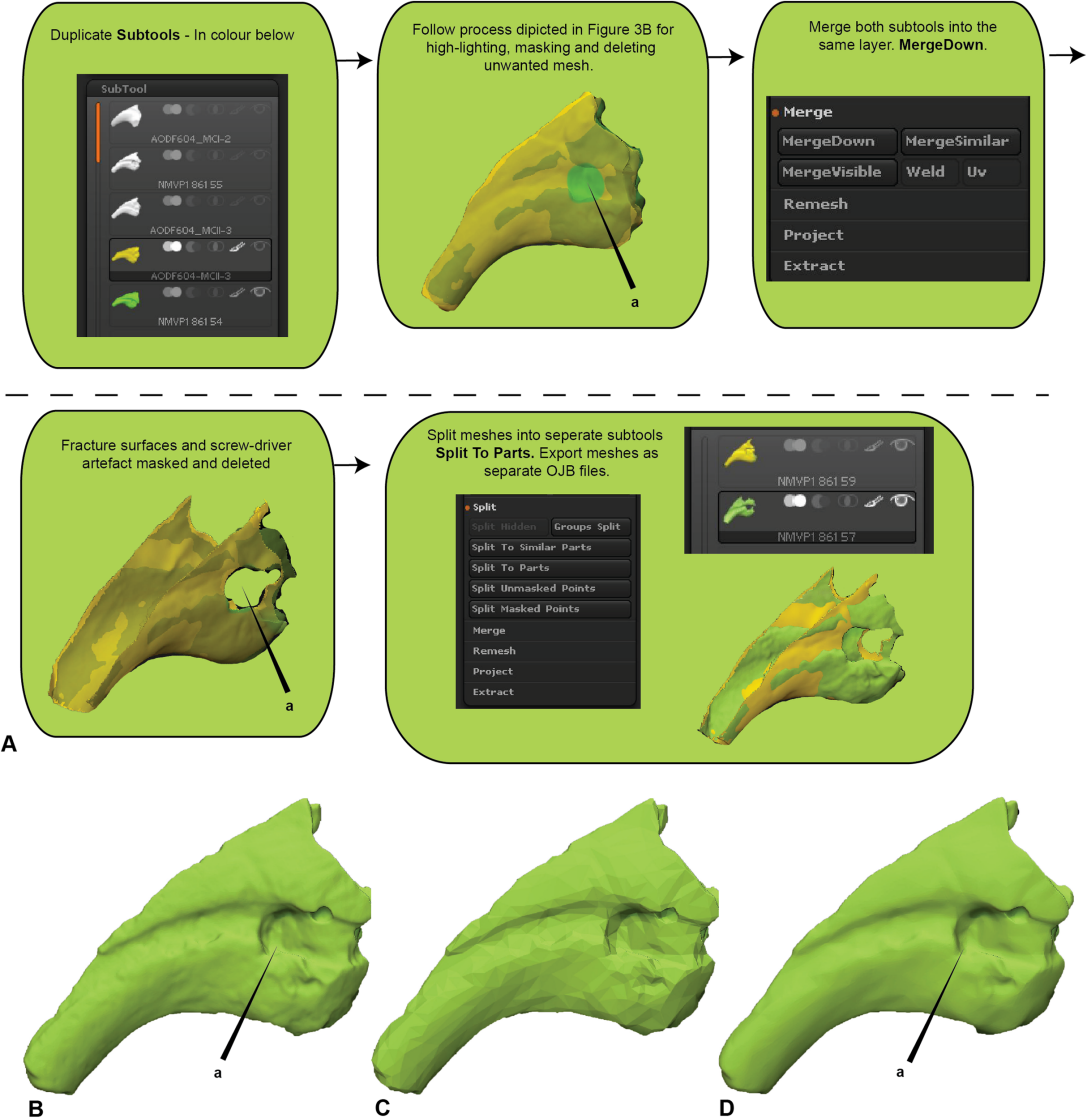
Zbrush surface removal and mesh resolution alteration. (A) Removal of undesired surfaces. To reduce to the resolution of a mesh, activate the target subtool. Along the top menu, select Decimation master from the Zplugin menu option. Decimation master has its own submenu, from where Pre-process current prepares the mesh for reduction. Upon completion, % of decimation is used to select the amount of mesh decimation. Decimate Current reduces the mesh. This process can be repeated until the desired resolution is acquired. Although not recommended, resolution increases is achieved through Divide from the Geometry menu. Increasing resolution can lead to loss of surface detail, as exemplified by our target specimen: (B) original resolution, (C) decimated resolution, (D) and re-increased resolution.

Initially, the fractured surfaces of NMV P186153 were replicated on the *A.wintonensis* manual phalanx II-3 by utilising the projection tools in zbrush (Fig. 2) to simulate equal fragments for pairwise comparison. However, as we later demonstrate, such surface replication inflates the similarity between the scans. Subsequently following the initial scaling and alignment, the regions not being compared were deleted prior to commencing pairwise comparison. These included major fractured surfaces, diagenetic and preparation artefacts (Fig. 4). This is an optional step and, if no further processing is needed, pairwise comparison can be initiated.

#### Resampling (Optional)

To maximise the information gleaned from pairwise comparisons, we recommend working at the highest resolution possible; a greater polygon count will provide a more faithful surface representation of anatomy, thereby increasing the power of the ICP algorithm. However, as scans are often obtained via different means, we recommend that comparisons should be performed between comparable resolutions, so as to limit any possible artefactual sources of variation. As a result, higher resolution scans should be reduced to match the polygon count of the lower resolution scan. Increasing the resolution of a low-resolution scan is not recommended, as it will not necessarily represent the original specimen. To demonstrate this, the scan of NMV P186153 was reduced to represent a low-resolution scan and then artificially increased to a comparable resolution as the original, resulting in substantial loss of detail from the original scan (Figs. 4B–4D).

#### Second registration

Following the removal/duplication of surfaces and resampling of resolution, a second round of registration is recommended. This provides the ICP algorithm the opportunity to re-scale and re-align using the more optimal data made available through the aforementioned mesh edits (steps outlined in Fig. 3).

### Comparative workflow

#### Pairwise comparison

**Figure 5:**
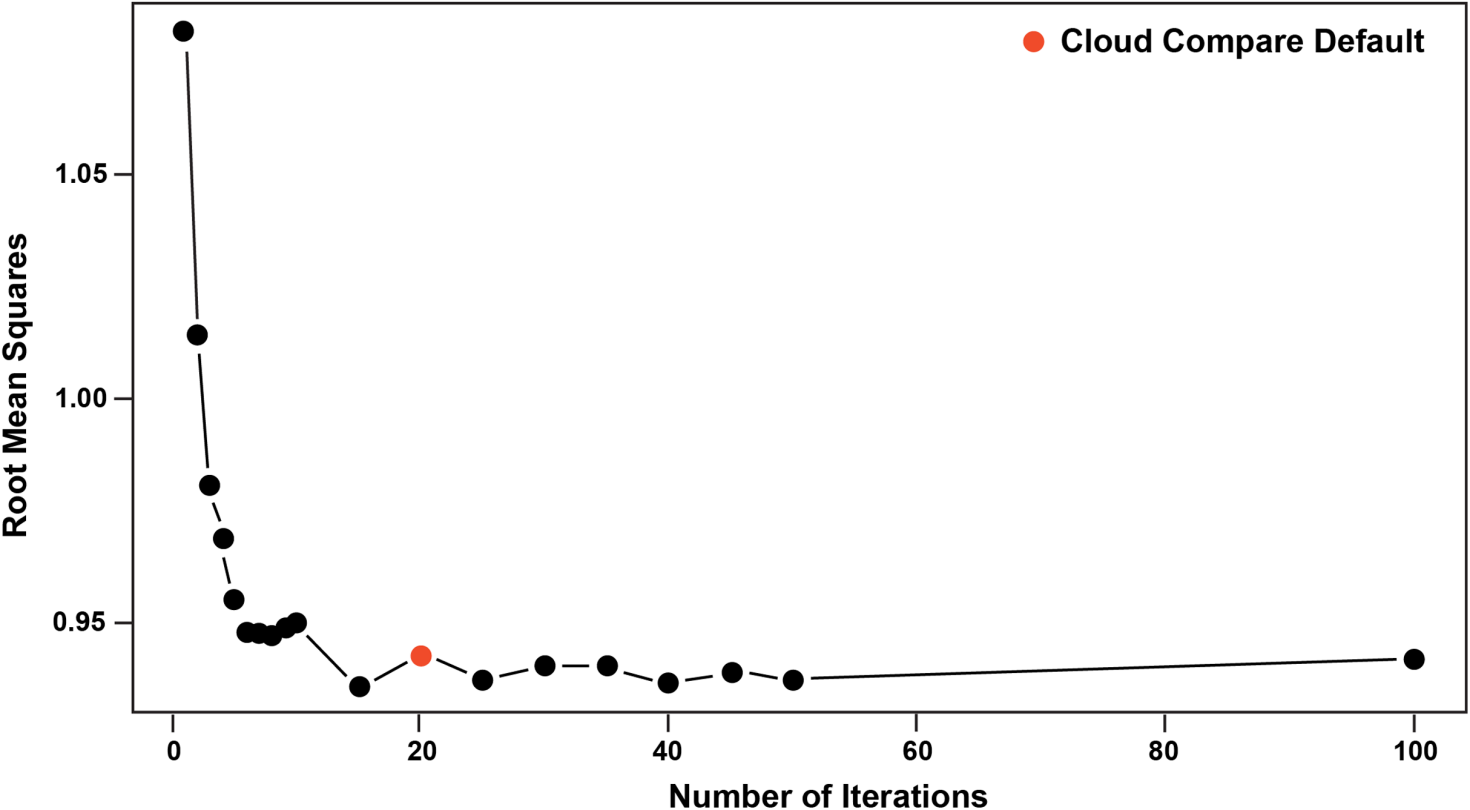
Iteration test confirming stabilisation of RMS at 20 iterations.

Pairwise comparisons, including a *Second Registration* between specimens, were achieved through the ICP algorithm (Besl & McKay, 1992; Pomerleau, Colas & Siegwart, 2015), which determines the transformation between a point cloud and a reference surface by minimising the root mean square point-to-point distance. The ICP algorithm aligns a target scan to a reference by (1) selecting close pairs of points between scans and calculating their distance as the mean squared Euclidean distance, (2) calculating the translation and rotation matrix needed to minimise the distances, (3) applying the transformation matrix to the target scan and recalculating the distances. This process is then repeated (iterated) using the new set of distances until convergence is reached and alignment is done (Besl & McKay, 1992; Chetverikov et al., 2002). The algorithm results in a vector of minimised distance values between the meshes that are then visualised as a distribution and described by standard statistics (i.e. mean, standard deviation, and range). We implement ICP through the open-source software Cloud Compare (CC) version 2.9.1 GPL software 2020. The default number of iterations in CC is 20, however, we explored this value in further detail by varying the number of iterations from 5 to 100 in increments of 5 and visualising its effect on the root mean square (RMS) point-to-point distances. The iteration test demonstrated that RMS stabilised at approximately 17 iterations, justifying the use of the default in CC (i.e. 20 iterations), which we subsequently used for the remainder of our analyses (Fig. 5).

**Figure 6:**
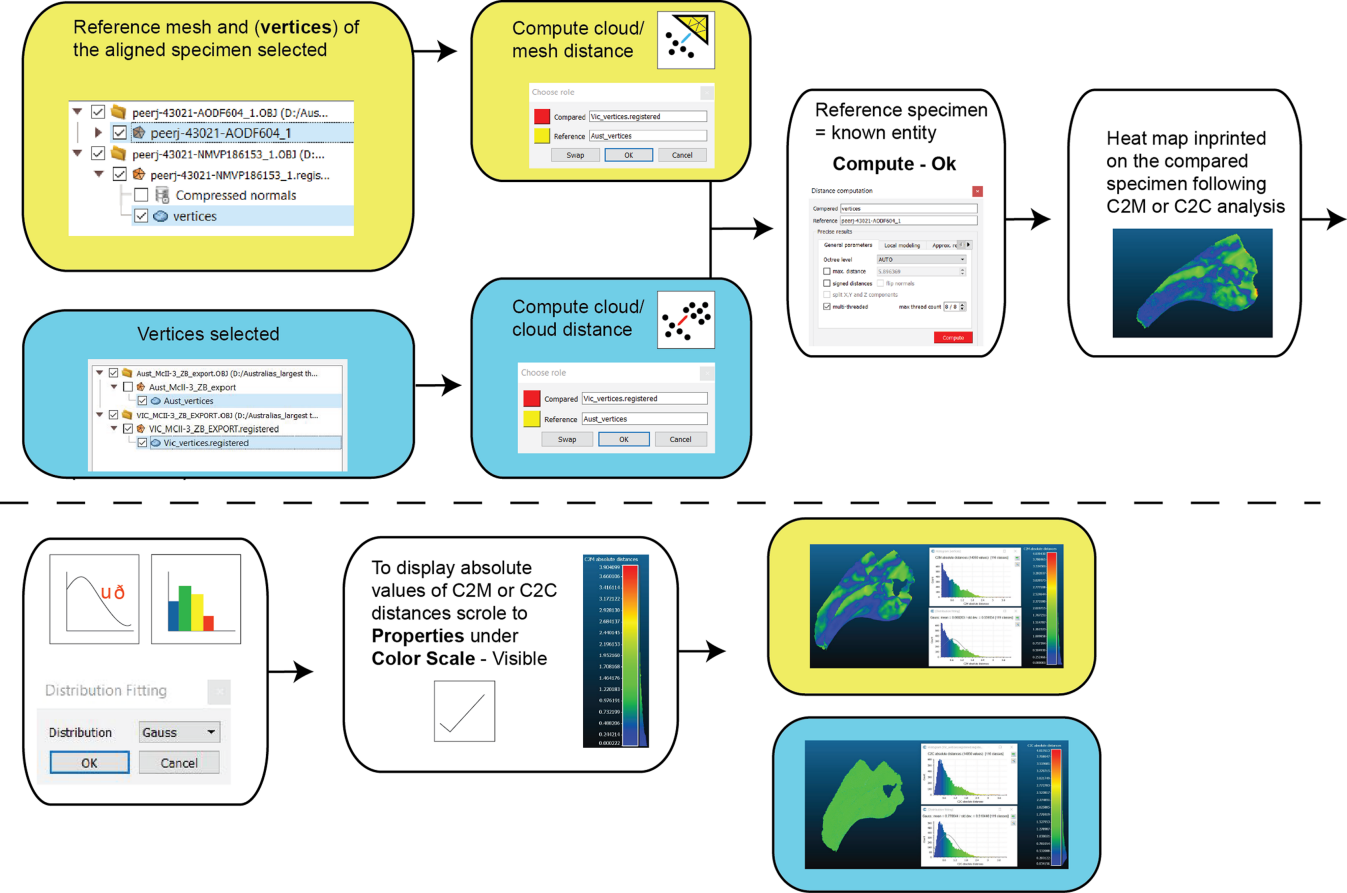
Workflow of cloud compare to compute C2M and C2C analysis and generate corresponding histograms and heat maps.

Two types of pairwise comparisons were carried out in CC: cloud to mesh (C2M) and cloud to cloud (C2C). In both cases, the final pairwise calculation was computed without the final automated ICP alignment (*Manual Registration*) and with the final automated ICP alignment (*Second Registration*) to assess whether that automation improved the initial manual registration. Point clouds (vertices) of both the reference and compared meshes are automatically created when opened in CC. The number of these points is based on the resolution of the scans. The distance between C2M or C2C is computed as the absolute Hausdorff distance (also called Pompeiu–Hausdorff distance) (Rockafellar & Wets, 2005) and projected onto the target specimen using a ‘heat-map’. The colder colours depict low distance values (i.e. target specimen is a close match to the reference specimen) and progressively warmer colours depict increased distances between the specimens (i.e. greater variation; Fig. 6). The distribution of the absolute distances are also visualised through a histogram. Our initial comparison included replicated artefactual surfaces of the compared specimen onto the reference specimen, whereas subsequent comparisons compared the specimens after the artefactual surfaces were removed (Figs. 4 and 6).

## Trial Results

The outcomes of the pairwise analysis are achieved through the set of workflows outlined in the previous sections. The following analyses are ancillary to the previous results of replicating surfaces (Analysis 1), deleting undesired surfaces (Analysis 2), and contextualising the results using a demonstrably different reference specimen (Analysis 3). In addition, each set of analyses demonstrate the effects of employing a final ICP alignment, as part of the *Second Registration* step following mesh editing; and using either the C2M and C2C comparison approaches. It is important to note that, due to the iterative approach employed by the ICP algorithm, each run will produce slightly different absolute distance values. However, results from such replications were found to be marginal and outcomes are relatively consistent.

### Analysis 1: artefactual surfaces replicated

**Figure 7:**
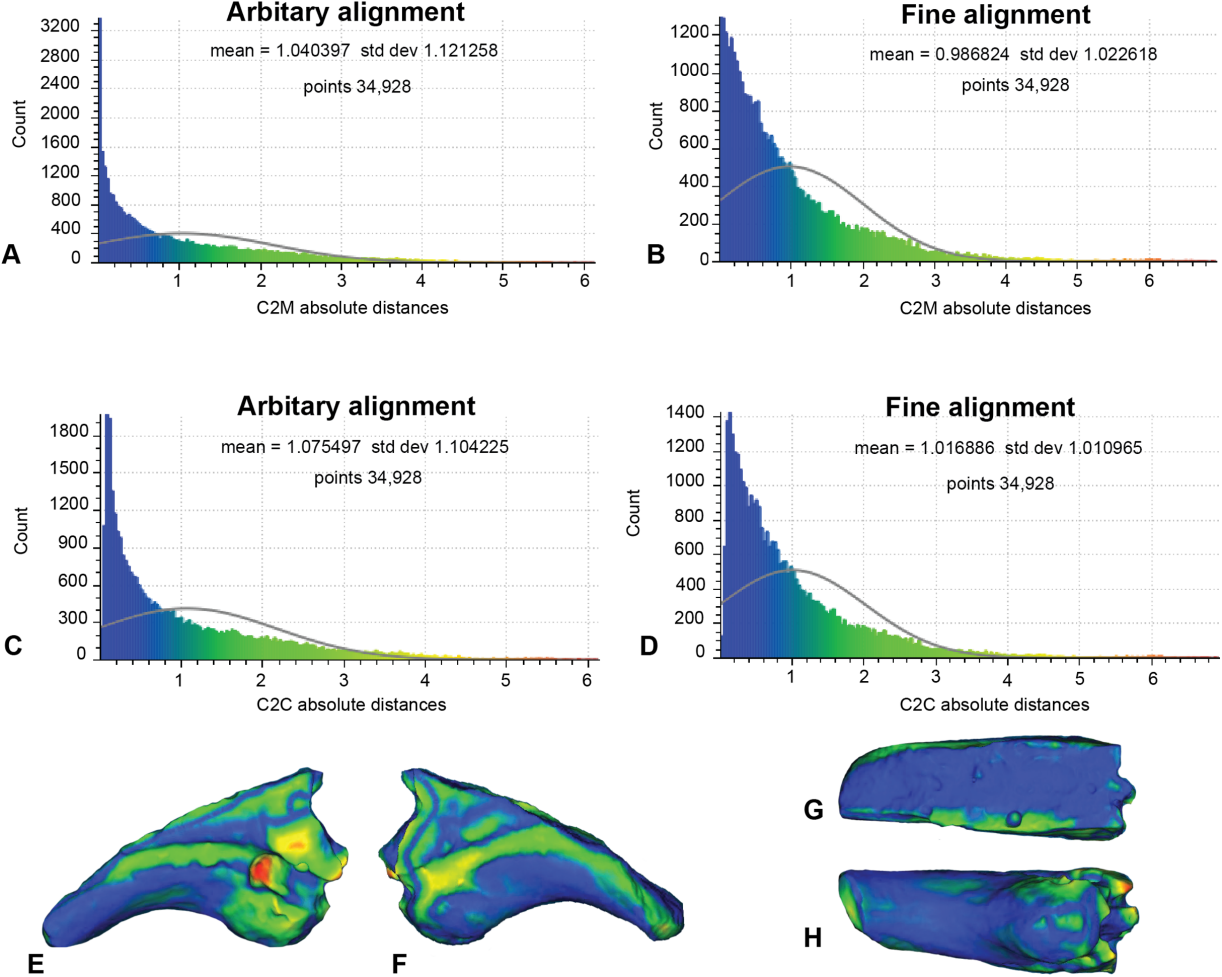
Replicated fractured surface of NMV P186153 projected onto the replicated equivalent of fragment of AODF604 MCII-3 (Analysis 1). (A) C2M arbitrary alignment. (B) C2M ICP fine alignment. (C) C2C arbitrary alignment. (D) C2C ICP fine alignment. Pairwise heat map of C2M depicting variation between the compared specimens imprinted on the compared specimen (NMV P186153) in: (E) Lateral. (F) Medial. (G) Dorsal fracture. (H) Ventral.

In this analysis, the fractured proportion of target specimen (NMV P186153) were projected onto the AODF604 MCII-3 to assess their effect if replicated (Fig. 7; Table 1). These scans comprise of 34,928 comparable points on which to calculate distances. The initial C2M analysis generated a range of distance values between 0 and 8.03, with a mean distance (MD) of 1.04 and standard deviation (SD) of 1.12 (Fig. 7A). The implementation of ICP (Fig. 7B) generated a slightly more constrained range, between 0 and 7.56 (MD = 0.99, SD = 1.02). In comparison, the initial C2C analysis produced a range between 0 and 8.03 (MD = 1.08, SD = 1.10; Fig. 7C), whereas with the fine alignment (ICP) the range was reduced to between 0 and 7.56 (MD = 1.02, SD = 1.01; Fig. 7D).

**Table 1:** Outcomes of Analyses 1 to 3.

Analysis	Parameters				Results		
Analysis	Removal	Comparison method	ICP	Reference	Mean distances	Standard deviations of distances	Number of distance values
Analysis 1	no	C2M	no	MCII	1.040397	1.121258	34,928
	no	C2M	yes	MCII	0.986824	1.022618	34,928
	no	C2C	no	MCII	1.075497	1.104225	34,928
	no	C2C	yes	MCII	1.016886	1.010965	34,928
Analysis 2	yes	C2M	no	MCII	0.927858	0.647785	14,050
	yes	C2M	yes	MCII	0.668333	0.559787	14,050
	yes	C2C	no	MCII	0.974374	0.613839	14,050
	yes	C2C	yes	MCII	0.771036	0.511375	14,050
Analysis 3	yes	C2M	no	MCI	2.130718	1.874513	12,780
	yes	C2M	yes	MCI	1.634286	1.351795	12,780
	yes	C2C	no	MCI	2.231208	1.814349	12,780
	yes	C2C	yes	MCI	1.739542	1.294163	12,780

### Analysis 2: artefactual surfaces removed

**Figure 8:**
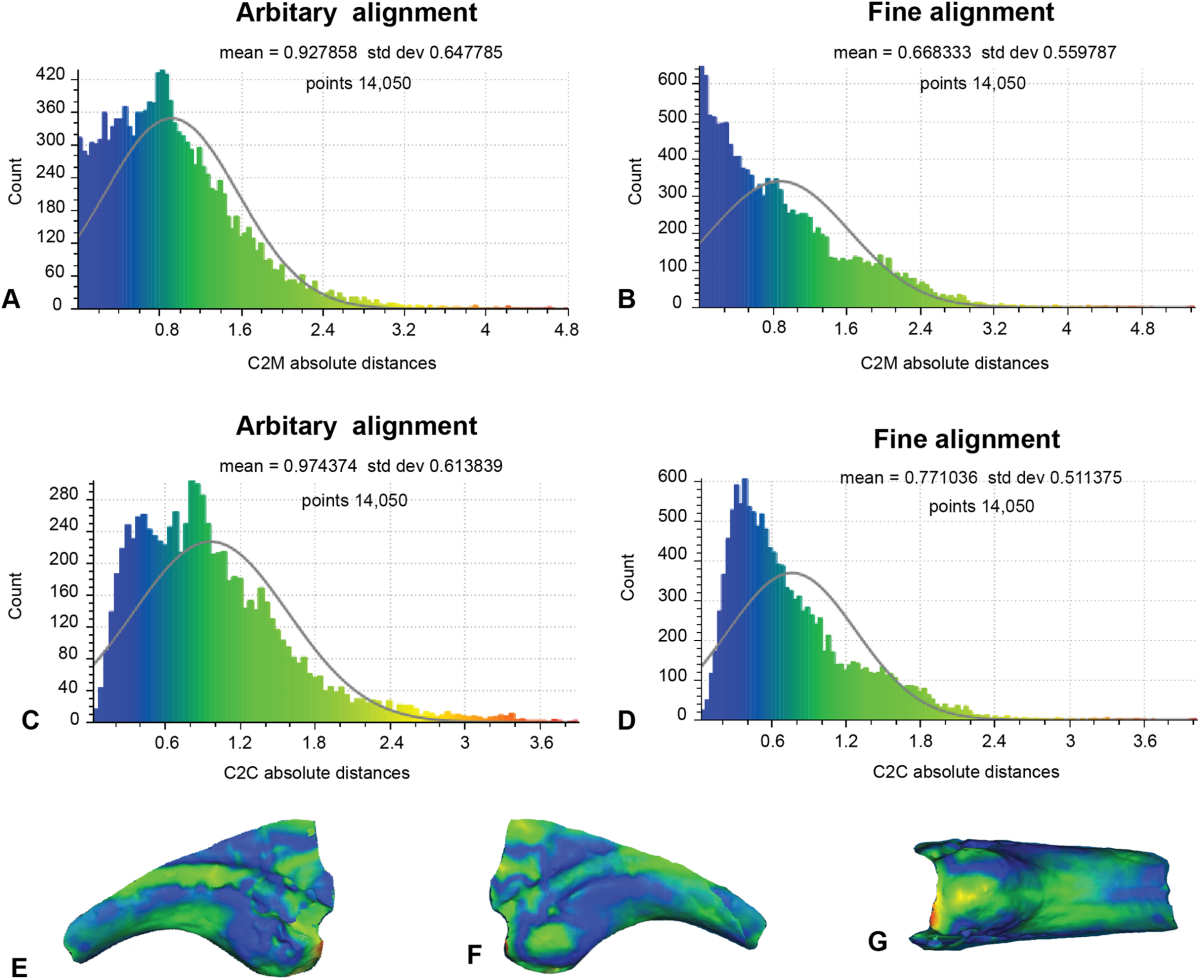
Fractured surfaces and excavation artefact of NMV P186153 removed with the corresponding margins also removed in AODF604 MCII-3 (Analysis 2). (A) C2M arbitrary alignment. (B) C2M ICP fine alignment. (C) C2C arbitrary alignment. (D) C2C ICP fine alignment. Pairwise heat map of C2M depicting variation between the compared specimens imprinted on the compared specimen (NMV P186153) in: (E) Lateral. (F) Medial. (G) Ventral.

This analysis excludes non-biological artefactual surfaces (Fig. 8; Table 1). As such, only 14,050 distances could be completed for analysis 2. The arbitrary C2M analysis generated distance values ranging between 0 and 5.42 (MD = 0.93 SD = 0.65; Fig. 8A). Implementation of ICP constrained the distance values to between 0 and 4.04 (MD = 0.67, SD = 0.56; Fig. 8B). The arbitrary C2C analysis distance values range between 0 and 4.80 (MD = 0.97, SD = 0.61; Fig. 8C), whereas the implementation of ICP constrained the distance values to between 0 to 4.02 (MD = 0.77, SD = 0.51; Fig. 8D).

### Analysis 3: comparison with an alternate reference specimen

**Figure 9:**
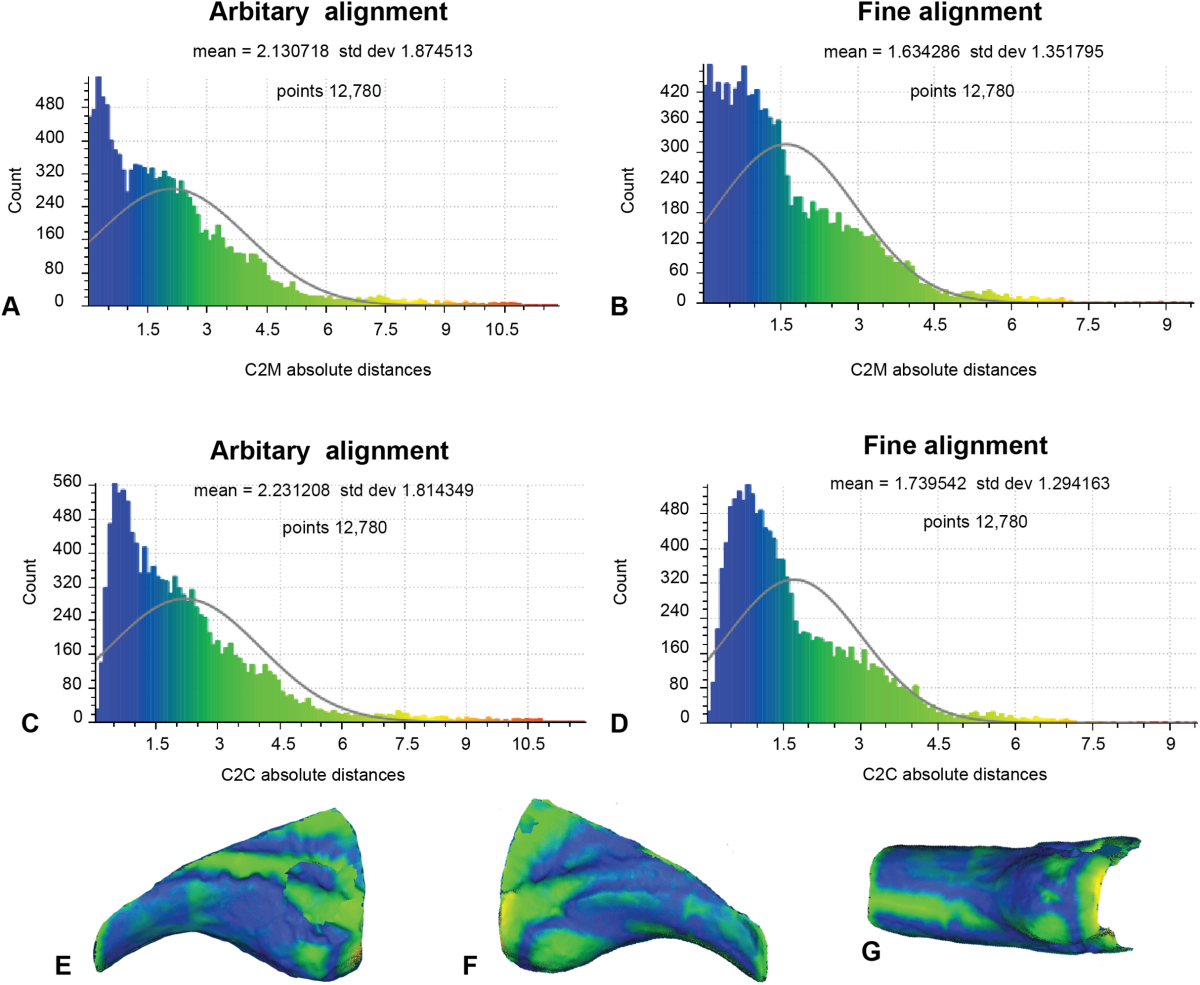
Fractured surfaces and excavation artefact of NMV P186153 removed with the corresponding margins also removed in AODF604 MCI-2 (Analysis 3). (A) C2M arbitrary alignment. (B) C2M ICP fine alignment. (C) C2C arbitrary alignment. (D) C2C ICP fine alignment. Pairwise heat map of C2M depicting variation between the compared specimens imprinted on the compared specimen (NMV P186153) in: (E) Lateral. (F) Medial. (G) Ventral.

Our earlier morphological descriptions identified distinct variation between our target specimen (NMV P186153) and our alternate reference specimen (AODF604 MCI-2) (Fig. 1). Accordingly, to better contextualise the results of analysis 2, we implemented the same approaches to the alternate reference specimens (Fig. 9; Table 1). Here, the number of vertices generated for comparison was 12,780, owing to the slightly reduced resolution of the scan obtained from AODF604 MCI-2. The arbitrary C2M analysis produced distance values ranging between 0 and 11.87 (MD = 2.13, SD = 1.87; Fig. 9A). The implementation of ICP constrained the range of values to 0 and 9.35 (MD = 1.63, SD = 1.35; Fig. 9B). The arbitrary C2C analysis generated a range of values between 0 and 11.87 (MD = 2.23, SD = 1.81; Fig. 9C). The implementation of ICP slightly constrained the range of values to between 0 and 9.28 (MD = 1.74, SD = 1.29; Fig. 9D).

## Discussion

### Interpretation of results/final workflow

The target specimen used in this study, NMV P186153, was originally described as sharing a close affinity with Megalosauroidea or Allosauroidea and was figured next to a near complete manual phalanx I-2 from the allosauroid *Chilantaisaurus tashuikouensis* (IVPP V.2884.2, Benson & Xu, 2008; see Fig. 15A-D in Benson et al. (2012)). At the time of description, only one partial Australian theropod skeleton was known, that of *Australovenator wintonensis* (Hocknull et al., 2009). The manual phalanges, MCI-2 and MCIII-4, that were reported in the initial description of *Australovenator* did not match NMV P186153; however, subsequently prepared elements of the holotype included a right MCII-3, which is distinctly similar to NMV P186153 (Fig. 1; see also Figs. 2 & 20 in White et al. (2012)).

Apart from its initial qualitative comparison, it is evident that a quantitative approach that digitally compares NMV P186153 to the unguals of *Australovenator*, such as through the pairwise comparisons presented here, would be beneficial. Our quantitative approach computes the absolute distances between two scans and offers a measure of fidelity between target and reference specimens. Distance values are then evaluated through standard statistics, such as mean and standard deviation, whereby larger mean and standard deviation values reflect greater differences and, conversely, values approaching zero reflect increased similarity. Such an approach would be advantageous to the broader palaeontological community, especially when considering highly fragmentary fossils that are difficult to include within larger sample sizes, and used to support standard descriptive comparisons between specimens.

Although we remain agnostic about the specific affinities of NMV P186153, our approach serves to support the previously noted similarities between NMV P186153 and MCII-3 of *Australovenator* (AODF604; Fig. 8; Table 1). In particular, we note that comparisons with MCI-2 lead to consistently significantly higher mean and standard deviation of distance values relative to comparisons with MCII-3 (Tables 1 and 2). Visually, variation ‘hot spots’ between our showcase specimens are concentrated proximally, both dorsal and ventral of the blood grooves, especially on the lateral surface (Figs. 7–9). The ventral ‘hot spot’ likely reflects the relatively larger flexor tubercle of NMV P186153 compared with AODF604 MCII-3 (Fig. 8), which is distinctly more ‘bulbous’ in shape compared to AODF604 MCI-2 (Fig. 9). Isometric size was by-in-large accounted for during the scaling process of our workflow and so the variation in flexor tubercle size may reflect positive allometry of this structure in *A. wintonesis*. However, without a more complete growth series, such inferences are speculative at this time. In comparison, ‘hot spots’ comparing NMV P186153 with AODF604 MCI-2, mark differences in the relative position of the blood groove, which is more dorsally located on MCI-2 (Fig. 9). Admittedly, the identification of these differences did not require pairwise comparisons (White et al., 2012), but outcomes of the comparisons with MCI-2 provide important quantitative and graphical context to the comparison with the preferred reference specimen (AODF604 MCII-3).

**Table 2:** Outcomes of the non-parametric analysis of variance of the pairwise trials (Table 1).

	df	SS	MS	*R*^2^	*F*	*Z*	*P*
Removal	1	0.075661	0.075661	0.024661	5.271181	1.155349	0.066
CM	1	0.014709	0.014709	0.004794	1.024744	0.50228	0.34
**ICP**	1	0.203619	0.203619	0.066368	14.18576	1.532015	0.013
**Reference**	1	2.413573	2.413573	0.786691	168.1496	2.68352	0.001
Residuals	7	0.100476	0.014354	0.03275			
Total	11	3.068004					

**Note:**

Row key as per parameter key in Table 1. Bolded parameters denote significant *p* values at a threshold <0.05. Column key: df, degrees of freedom; SS, sums of squares computed using a type II hierarchical approach; MS, mean squares, *R*^2^, coefficients of determination; F, *F* statistics; Z, effect sizes; *P*, significance *p* values.

On a more general level, comparisons between the analyses using a non-parametric analysis of variance demonstrate that our outcomes are primarily driven by the choice of reference specimen (*R*^2^ =0.787, *p* = 0.001; Table 2), which is unsurprising given the aforementioned distinctly different morphologies between AODF MCI-2 with NMV P186153 (e.g. Figs 1, 8 and 9; White et al., 2012), but underscore the importance of making multiple pairwise comparisons. Also significant was the implementation of a final ICP to scale and closely align the scans following mesh modifications (*R*^2^ = 0.066, *p* = 0.013; Table 2). Implementing a final ICP consistently led to lower mean distances and more constrained ranges (Table 1). Interestingly, whether broken/undesirable surfaces are replicated or removed did not lead to significantly different pairwise comparison outcomes (*R*^2^ = 0.025, *p* = 0.066; Table 2). However, as noted in the histograms (Figs. 7 and 8), the replication of fractured surfaces leads to artificially lower modalities and leptokurtic distributions suggesting that removal of such surfaces are likely to generate a more faithful representation of variation between scans. Finally, choice of comparison approach, whether C2M and C2C, had very low explanatory power (*R*^2^ = 0.005, *p* = 0.34; Table 2), suggesting that either is viable.

**Figure 10:**
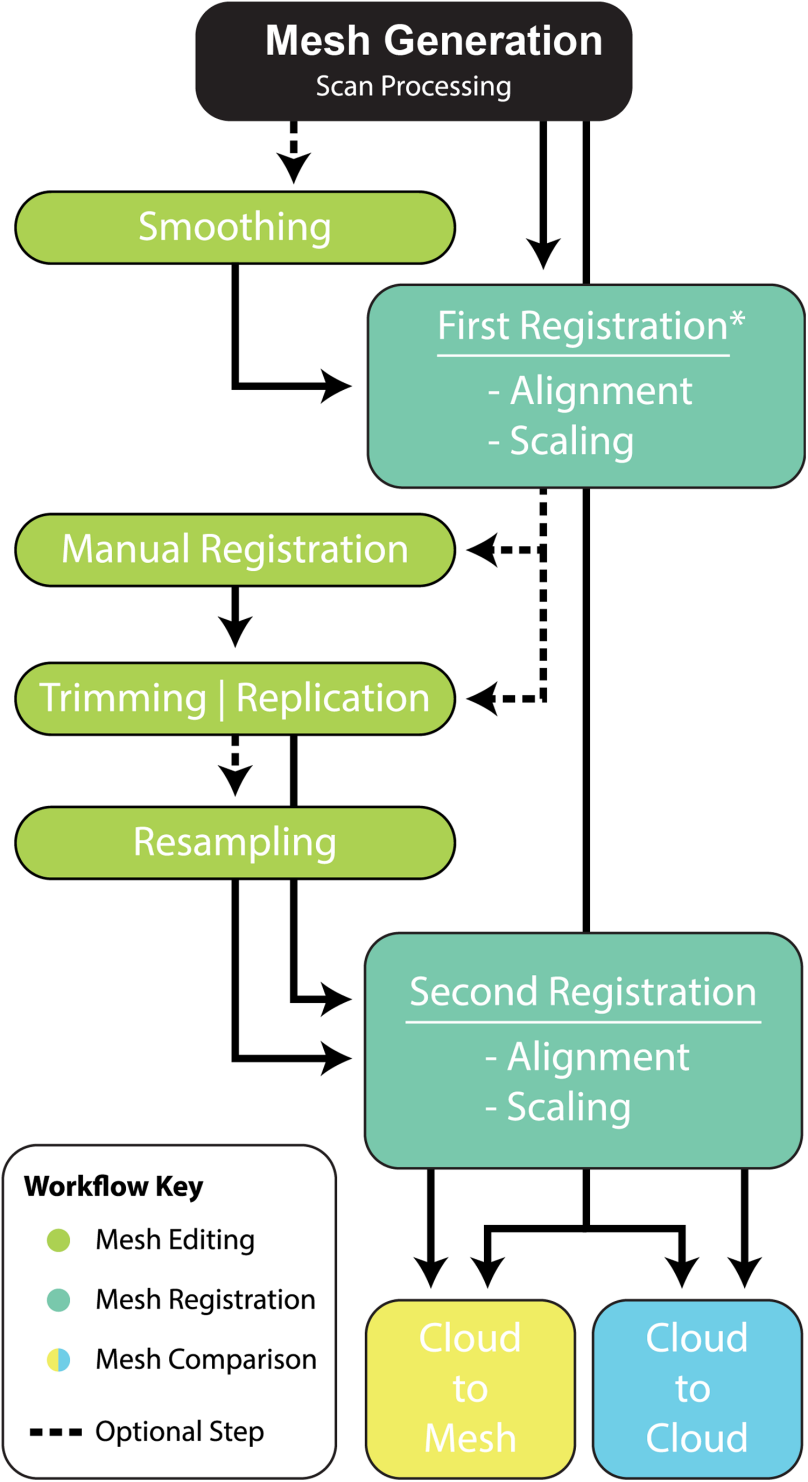
Workflow of implementing a pairwise comparison of specimens.

Given these results, we provide the following best-practice recommendations and final workflow (Fig. 10):

1. Scan preparation: 3D models of specimens should be generated at as high a resolution as possible to maximise the number of points available to the ICP algorithm to align and compare specimens. It is not recommended, however, that 3D models be generated at a resolution beyond that provided by the scanner (Fig. 4).
2. Scaling/Alignment: The initial scaling and alignment processes (i.e. First Registration) require the determination of primary homology between specimens (De Pinna, 1991), after which select homologous landmarks (at least 3) are chosen to objectively scale and align the models. However, this objective registration approach needs subsequent visual inspection as it may prove ineffective; manual scaling and/or alignment can rectify such discrepancies and later re-adjusted mathematically through a final ICP fine alignment scaling (i.e. Second Registration; Fig. 2).
3. Scan comparability: Following our experiments, it is evident that replicating artefactual surfaces leads to leptokurtic distributions (Fig. 7), indicative of an over-inflation of modal values driven by the identical replicated surfaces. We support the omission of such artefacts and use of open 3D models for subsequent comparisons.
4. Pairwise comparison: Final alignment carried out by ICP prior to the final comparative analysis is strongly recommended. In all our permutations, ICP-aligned comparisons generated lower mean and standard deviation values suggesting it consistently achieved better alignment between specimens than the initial alignment via selecting homologous points and, if required, further manual scaling and alignment. Furthermore, ICP alleviates some of the subjectivity incurred during manual registration. Mapping of variation ‘hot spots’ onto the target specimens provides a useful representation of variation, which can be used to support qualitative descriptions. Importantly, we discovered that in analysing absolute values the mean and standard deviation of the distance values can be used as a measure of fidelity between scans, whereby lower means and standard deviations indicate greater overall similarity (Figs. 7–9; Table 1).

### Practicality and utility

Geometric morphometrics, which applies ordination techniques to outline, surface, and/or landmark data, is a powerful tool with which to explore patterns of morphospace occupation, given a specific dataset (see Zelditch, Swiderski & Sheets (2012) and Adams, Rohlf & Slice (2013) for a review on the topic). This approach is often applied to the fossil record to investigate, for example, macroevolutionary dynamics (Bazzi et al., 2018; Piras et al., 2014; Polly, 2003; Polly & Head, 2004). More relevant to our goals, however, morphometrics can be used to assess taxonomic hypotheses, given an appropriately large data set that attempts to accommodate for intra- vs. interspecific variability (Campione & Evans, 2011; Hedrick & Dodson, 2013; Polly, 1998; Polly, Le Comber & Burland, 2005). Other viable approaches for taxonomic identification include neural networks and a number of computational algorithms that intend to automate species identification based on a priori built training datasets (Ripley, 1994; Behnke, 2003; Cope et al., 2012; Hsiang et al., 2019; Du et al., 2010), collectively referred to as computer aided taxonomy (CAT) (Cope et al., 2012; Du, Wang & Zhang, 2007; MacLeod, O’Neill & Walsh, 2016). However optimal, these dataset-driven approaches depend greatly on the nature and size of the training set and are ideally suited for complete specimens (Du, Wang & Zhang, 2007).

To our knowledge, the utility of pairwise comparison methods, such as ICP, in the absence of large comparative data sets has not been explored for fossil data. Therefore, its application to incomplete specimens and fundamental palaeontological problems, such as specimen identification, seem evident here. The pairwise comparison workflow presented here offers the possibility to quantitatively evaluate the morphology of fragmentary fossils that might otherwise be ignored, and support qualitative anatomical observations used for taxonomic assignments. Uniquely, this approach does not require large sample sizes, can be applied to a select few exemplars, and is particularly advantageous when considering fragmentary fossil records, such as those of Australian Cretaceous dinosaurs (White et al., 2020). It should be emphasised, however, that an isolated pairwise comparison (i.e. with no other additional comparative contexts) neither supports nor rejects a taxonomic assignment. Pairwise comparisons can provide quantitative support for qualitative observations but cannot replace the power of data-driven approaches such as geometric morphometrics and CAT tools. Rather, the observations gleaned from the quantitative pairwise comparisons, such as those demonstrated here, serve to quantitatively support and expand on otherwise purely qualitative anatomical descriptions. In our case study, our two comparisons serve to support that NMV P186153 likely pertained to digit II rather than I.

Our methodology is showcased to visualise and quantify pairwise comparisons between specimens in an effort to assist in the identification of fragmentary fossils. However it could be employed to demonstrate a number of other descriptive scenarios with a potential benefit to other biological and geological questions. These could include, but are not limited to: the assessment of specimen deformation caused by taphonomic processes; and the three-dimensional representation of variation along a continuum, such as an ontogenetic growth series or along a canonical axis of variation.

#### Study of deformation

The ability to quantify the amount of deformation a fossil has sustained is crucial for studies of taxonomy, ontogeny, and biomechanics (Arbour & Currie, 2012; Hedrick & Dodson, 2013; Tschopp, Russo & Dzemski, 2013). As described in our workflow, we have attempted to remove the extrinsic sources of variation, by excluding broken region. However, our showcase specimens are not diagenetically altered, preserving the original shape of the bones. Our pairwise approach could be used to study the effects of three-dimensional deformation, especially when considering retrodeformation procedures.

Retrodeformation—the process of virtually deforming a fossil to its perceived original three-dimensional form (Williams, 1990)—has seen extensive use among palaeontologists, including: plesiosaurs (Motani, Amenta & Wiley, 2005), snakes (Polcyn, Jacobs & Haber, 2005), early tetrapods (Molnar et al., 2012), primates (Ponce de León & Zollikofer, 1999, Gunz et al., 2009), and dinosaurs (Arbour & Currie, 2012; Tschopp, Russo & Dzemski, 2013; Tschopp & Mateus, 2013). The base requirement of retrodeformation is the ability to identify bilaterally symmetric landmarks. These identifications can be problematic as the original undeformed morphology is unknown (Tschopp, Russo & Dzemski, 2013). Asymmetry and symmetrical deformation such as compression was also found to be problematic for the retrodeformation process (Tschopp, Russo & Dzemski, 2013). One, yet-to-be explored possibility, is the use of 3D meshes and pairwise comparisons, such as the one presented here, to quantify and visualise the extent of bilateral asymmetries likely to have been the result of taphonomy. This process would require the identification of the bilateral axis and the mirroring of one side to match the other. However, once its extent is determined, asymmetrical variation ‘hot-spots’ could be then be used to identify where landmarks are needed prior to subsequent retrodeformation procedures.

#### Study of variation

The variation identified through our pairwise approach need not be taxonomic, and the technique could be extended to visualise regions of intraspecific variation, whether between juvenile and adult or male and female members of the same species. Much like the Procrustes algorithm implemented in modern geometric morphometrics (Bookstein, 1991), the automated and manual registration procedures outlines above serve to remove the effects of isometric size. Assuming the specimens pertain to the same species and same anatomical region, any remaining variation noted through pairwise comparison must then be the result of intraspecific variation, whether ontogenetic or sexual.

One alternate use of the ICP-based pairwise approach presented here could be the graphical representation of morphological continua across canonical axes of variation (Claude, 2008; Márquez et al., 2012; Sansalone et al., 2020). Given a three-dimensional reference landmark configuration (e.g. the mean configuration following principal component ordination), target configurations at theoretical values along the canonical axes, whether at the extreme of the axes or at given intervals (Olsen, 2017), could be visualised using ICP. The outcome of this approach would be a 3D depiction of variation along the axes of variance, akin to that generated by Sansalone et al. (2020), which similarly depicted variation in 3D as a heat map through a processes of interpolation. The ICP algorithm for landmarks was implemented as part of the R package *Morpho*, via the function *icpmat* (Schlager, 2017) but, to our knowledge, no such implementation yet exists for meshes in R.

## Conclusions

The methodology and workflow explored in this study offers the possibility to quantitatively support fundamental but qualitative palaeontological observations aimed at the identification of fragmentary fossils that might otherwise be ignored or ambiguously assigned to taxonomic groups. Innovatively, this approach does not require the prior construction of large morphometrics data sets but depends on substantial 3D virtual manipulations of scans. The output generates both a visual and numerical representation of variation that can accompany descriptions and, given adequate context, permit the assessment of taxonomic and anatomical identification. Although this approach does not have the interpretive power of dataset-driven comparative methods, our study provides the basis for a fundamental tool for both anatomists and curators seeking to quantitatively support the identification of fragmentary specimens. Finally, the pairwise nature of this approach has evident implications to the study of non-taxonomic sources of variation, whether the result of taphonomy or ontogeny, and could be adapted to visualise variation along canonical axes of variation.

## Supplemental Information

10.7717/peerj.10545/supp-1 Supplemental Information 1 NMVP186153.

10.7717/peerj.10545/supp-2 Supplemental Information 2 AODF 604 MCII-3 Fragment.

10.7717/peerj.10545/supp-3 Supplemental Information 3 NMVP186153 Open Shell.

10.7717/peerj.10545/supp-4 Supplemental Information 4 AODF 604 MCII-3 Open shell.

10.7717/peerj.10545/supp-5 Supplemental Information 5 NMVP186153 Open shell.

10.7717/peerj.10545/supp-6 Supplemental Information 6 AODF 604 MCI-2 Open shell.

## Additional Information and Declarations

### Data Availability

The following information was supplied regarding data availability: Data is available in the Supplemental Files and at Morphosource: https://www.morphosource.org/Detail/ProjectDetail/Show/project_id/1149. M85221-165644: DOI 10.17602/M2/M165644 M85221-165647: DOI 10.17602/M2/M165647 M85221-165648: DOI 10.17602/M2/M165648 M85222-165649: DOI 10.17602/M2/M165649 M85222-165650: DOI 10.17602/M2/M165650 M85222-165651: DOI 10.17602/M2/M165651.
